# Variability in the Labeling of Asthma among Pediatricians

**DOI:** 10.1371/journal.pone.0062398

**Published:** 2013-04-24

**Authors:** David Van Sickle, Sheryl Magzamen, Matthew J. Maenner, Julian Crane, Timothy E. Corden

**Affiliations:** 1 Department of Population Health Sciences, University of Wisconsin School of Medicine and Public Health, Madison, Wisconsin, United States of America; 2 Department of Medicine, Otago University, Wellington, New Zealand; 3 Department of Pediatrics, Medical College of Wisconsin, Milwaukee, Wisconsin, United States of America; University of Liverpool, United Kingdom

## Abstract

**Objective:**

Few studies have examined variability among physicians in the perception and interpretation of asthma symptoms. We report the results of a pilot study to investigate the variability of symptom description and diagnostic labeling and nomenclature among a group of clinicians using standardized audiovisual presentations of asthma.

**Methods:**

Practicing pediatricians in Wisconsin recruited from an electronic mailing list were shown the International Study of Asthma and Allergies in Childhood (ISAAC) video questionnaire online, and asked to describe the symptoms and signs they observed and suggest possible diagnostic labels for each presentation.

**Results:**

A total of 113 pediatricians (mean age = 43 years; 56% female) responded to ≥1 of the 5 video scenes. The number of practitioners who described the principal symptom(s) of asthma depicted in the 5 sequences ranged from 5.5% for Scene 5 (featuring both dyspnea and wheeze), to 100% for Scene 4 (featuring cough). The number who suggested label of ‘asthma’ as a possible cause of the presentations ranged from 69.7% for Scene 3 (featuring nocturnal wheeze), to 92.7% for Scene 2 (featuring exercise induced wheeze).

**Conclusion:**

There is important unexplained variation in the perceptions and labeling of asthma symptoms among pediatricians. These differences may influence the likelihood of diagnosis and the apparent prevalence of asthma. Many participants suggested that the ISAAC video be used in the education and training of pediatricians.

## Introduction

The lack of a standardized definition of asthma is an ongoing challenge for asthma epidemiology. Unlike Europe, Asia, and Australia, where asthma epidemiology is largely informed by the European Community Respiratory Health Survey (ECRHS) and the International Study of Asthma and Allergy in Childhood (ISAAC), investigation of asthma prevalence and asthma-related morbidity in the United States has been predicated upon national surveys in which the key indicator for asthma is report of physician diagnosis.[1]–[3] Report of diagnosis, rather than determination of prevalence by report/presentation of symptoms, poses two distinct challenges for epidemiologic research. Differences in diagnosed asthma may partially reflect differences in access to health services and diagnostic practice rather than differences in asthma prevalence. [4] Although studies in the US based on symptom data have confirmed that limited access to healthcare is tied to undiagnosed asthma in low-SES communities, [5], [6] little is known about the contribution of variations in asthma diagnosis to prevalence estimates. As a result, systematic differences in the perception of asthma symptoms and their clinical interpretation and labeling may play an important role in the apparent patterns of disease prevalence. [7] Artefactual changes in asthma prevalence may arise from changes over time in the perception and interpretation of symptoms, [8], [9] and differences in the proportion of the population with symptoms labeled as asthma.[10]–[12] Early population-based research suggested that asthma epidemiology is dependent on the diagnostic habits of physicians in the locale, as well as a measure of the prevalence of a specific syndrome. [13] Given acknowledged limitations in the label of asthma [14], [15] and accumulating evidence that physicians may vary in their classification of disease, [16] assumption of a uniform interpretation and application of the diagnostic label of asthma in these asthma questionnaires may lead to bias in interpretation of these data. In order to adequately interpret data collected from these national surveillance efforts, a valid methodology for reliably determining how physicians perceive and label asthma and asthma symptoms across geographic and clinical settings is required.

The objective of this pilot study was to examine variability in the nomenclature and labeling of asthma and its primary symptoms among a sample of pediatricians using the standardized audiovisual presentations of asthma of the International Study of Asthma and Allergies in Childhood (ISAAC) video questionnaire. This methodology allowed us to collect information about the perception and labeling of asthma by physicians in a manner that minimized bias introduced by the effect of language, culture, and interview technique, as well as patient mix and clinical setting.

## Methods

### ISAAC Audiovisual Questionnaire

The ISAAC video questionnaire (AVQ 3.0, © Otago University) was developed in the 1990s by the Wellington Asthma Research Group to be a standardized methodology for determining the prevalence of asthma symptoms in children. [17]–[19] To date, it has been used among hundreds of thousands of children in more than 40 countries to estimate the prevalence of asthma symptoms [20].

In the international version of the video, young adults from a variety of ethnic backgrounds can be seen and heard manifesting different symptoms of asthma during a set of five short (25 second) sequences. The sequences display a Caucasian female seated with moderate wheezing at rest; two Maori males, one who wheezes after exercise, and one who does not; an East Asian male waking at night with wheezing; a Caucasian female waking at night with coughing; and a South Asian female with a severe attack of asthma with wheezing and dyspnea. Further information about the development, validation and utilization of the video questionnaire has been published elsewhere [17]–[19].

### Study Population and Setting

In 2008, we recruited practicing pediatricians from the electronic mailing list of the Wisconsin chapter of the American Academy of Pediatrics (WI-AAP) to participate in the survey. Participants viewed the video scenes online and completed the survey electronically.

### Survey Instruments, Responses and Coding

The anonymous survey instrument (see Appendix S1) included questions on demographics (age, sex, race), training and education (board certification, major practice area), and practice characteristics (location, setting, average number of patients per day, insurance and Medicaid status of patients). No personally identifying information was collected by the survey.

Potential participants were instructed that that they would be taking a survey to evaluate their perceptions of respiratory symptoms. The terms ‘asthma’ and ‘ISAAC’ were never referenced in the recruitment materials or in the survey. Practitioners were not informed in advance that the video was an instrument used in asthma epidemiology, nor that the purpose of the study involved asthma in any way.

Participants were asked to describe all of the signs and symptoms they observed in each video scene and to suggest for each scene likely diagnoses or causes of the presentation. No further guidance or restrictions were placed on the number or nature of their responses to the questions. Single term responses, such as wheeze, were permitted. We included and retained all answers, and collapsed or aggregated only misspelled or derivations of single terms, such as wheeze and wheezing.

### Statistical Methods

For each video scene, we calculated p-values and odds ratios using Cochrane-Mantel-Haensel tests to determine the association between the description of symptom(s) and suggestion of the label of asthma. To assess the relationship between demographic, training, and practice variables, and suggestion of the label of asthma for each of scenes, participants who provided diagnostic labels for all five scenes were categorized into those who suggested the label asthma for all five scenes and those who did not. We conducted a bivariate analysis with all demographic, training and practice variables collected from the survey, and included variables significant at the p<0.15 level in a multivariate logistic regression model. All statistical analyses were conducted with SAS Version 9.2 (SAS, Cary, NC, USA).

### Ethical/Human Subjects Review

This research was reviewed and approved by the Executive Committee of the Wisconsin Chapter of the American Academy of Pediatrics, the Health Sciences Institutional Review Board at the University of Wisconsin – Madison, and the Institutional Review Board at the Medical College of Wisconsin. A small donation was made to the Wisconsin Academy of Pediatrics Foundation for each participant who completed the survey and video questionnaire.

## Results

### Study Population

The survey was electronically mailed to 962 WI-AAP registered clinicians. A total of 116 (12%) clinicians completed the survey. Three participants reported familiarity with the video and were excluded from the primary analysis. Participants averaged 43 years of age (range: 26–72) and reported a mean of 13.3 years in practice (range: 0–40). Table 1 summarizes the main demographic and clinical characteristics of the study sample. More than half of the respondents were female (56%), and general pediatricians (79%); a total of 18 sub-specialties were reported. A majority of respondents practiced in urban settings (54%) in a hospital or clinic (56%). Clinicians from 20 (28%) counties in Wisconsin participated in the survey with clusters of responses from the major population centers of the state. Data on select attributes of the study population were available through the WI-AAP database. Mean age of WI-AAP members was also 43 years (range: 20–83). Overall, 95% of WI-AAP members listed their practice in the state. Total membership represented 46 counties, with the majority of physicians located in the population centers [Milwaukee County (28%) and Dane County (19%).] There was no significant difference between proportion of total members and survey respondents from Milwaukee County (p = 0.10) but a significantly higher proportion of respondents from Dane County were included in the study sample compared to the study population (p = 0.03). There were no significant differences in gender (55% female v. 56% female, p = 0.92) or percent board certified (74% v. 79%, p = 0.56) between the study population and study sample, respectively. The study population had a significantly lower percentage of current pediatric residents compared to the study sample (3% v. 19%, p<0.001.).

**Table 1:** Demographic and clinical practice characteristics of survey respondents (n = 113).

Characteristic	N	%
Sociodemographic		
Age, in years (mean)	43	
Female	63	56.2
Race		
White	98	86.7
Black	0	0
Latino	2	1.8
Asian	7	6.2
Refused	4	3.5
Clinical practice		
Location		
Rural Practice	11	9.7
Suburban	40	35.4
Urban	61	54.0
Board Certified	89	78.8
General Pediatrics	79	69.9
Pediatrics Resident	22	19.5
Type of Practice#		
Group	53	46.9
Hospital/Clinic	63	55.6
Medical School	28	24.8
Pct Medicaid		
0–30%	62	54.9
>30%	50	44.3
Pct Uninsured		
0–10%	82	72.6
>10%	28	24.7

# Practice categories are not mutually exclusive; respondents able to choose more than one.

### Symptom Description

Practitioners reported a wide range of symptoms in each of the five video sequences. A total of 143 different symptoms were suggested by practitioners who completed the survey. The mean (±SD) number of distinct symptom labels suggested by the entire group of practitioners for the five scenes was 29 (±5.0), with a range of 25–37. Individually, practitioners reported an average of 3 (±2.8) symptoms per scene. Wheeze was the most frequent symptom reported for four of the five scenes [range: 56% (Scene 5) - 91% (Scene 2)] and the most common symptom identified overall. Cough was noted by the second largest number of practitioners, largely because it was the principal characteristic in Scene 4 (identified cough: 100%), and commonly identified even when it represented an incidental part of the presentation, as in Scene 1 (identified cough: 61%).

The number of participants who suggested the principal characteristic(s) of the scene ranged from 5.5% for Scene 5 (featuring dyspnea and wheeze, both required) to 100% for Scene 4 (nocturnal cough). The majority of survey respondents (64.5%) reported the principal characteristic for three of the five scenes; 3.5% of respondents described the principal characteristic for all five scenes.

### Disease Interpretation and Labeling

Overall, 70 diagnostic labels were used to describe the likely causes of the five presentations. The number of labels offered by practitioners ranged from 11 (for Scene 2, exercise-induced wheeze) to 26 (for Scene 3, nocturnal wheeze). The most common interpretations for the five scenes are presented in Table 2. Asthma was the most frequently suggested diagnostic label for all 5 scenes, which ranged from 70% for Scene 3 (nocturnal wheeze) to 93% for Scene 2 (exercise-induced wheeze.) Nearly half of all respondents (47.8%) suggested the label of asthma for all five scenes. Only two individuals did not suggest the term asthma for any of the five scenes. There were no suggestions of asthma sub-diagnoses (e.g. allergic asthma), with the exception of Scene 2, in which exercise-induced asthma, allergy-induced asthma and exercise-induced bronchospasm where both suggested, and classified as positive asthma responses.

**Table 2:** The most common diagnostic labels suggested for each of the five audiovisual scenes.

Scene 1	n	Scene 2	n	Scene 3		Scene 4	n	Scene 5	n
Asthma	102	Asthma or exercise induced asthma	88	Asthma	76	Asthma	81	Asthma	82
Bronchospasm	6	Exercise induced bronchospasm	21	Croup	17	Upper respiratory infection	12	COPD	18
Reactive airway disease (RAD)	5	Vocal cord dysfunction/paralysis	8	Upper airway obstruction	12	Pertussis, parapertussis, or whooping cough	11	Airway obstruction	5
Airway obstruction	3	Overexertion, out of shape, poor conditioning	5	Bronchospasm	6	Bronchospasm	9	Bronchospasm	5
Upper respiratory infection	2	Reactive airway disease (RAD)	3	Congestive heart failure	5	Reactive airways disease (RAD)	7	Reactive airways disease (RAD)	4
COPD	1	Exercise-induced wheeze	1	Reactive airway disease (RAD)	5	Gastroesophageal reflux	5	Pneumonia	3
Lower respiratory infection	1	Allergy-triggered asthma	1	Vocal cord dysfunction	4	Respiratory infection	4	Congestive heart failure	3
Unsure	1	Decreased cardiac reserve	1	Laryngotracheobronchitis	3	Postnasal drip	4	Emphysema	2
Expiratory issues	1	Fatigue	1	Gastro-esophageal reflux disease	2	Bronchitis	3	Vocal cord dysfunction	2
Croup	1	Lower airway obstruction	1	Paroxysmal nocturnal dyspnea	2	Sinusitis	3	Croup	2
Other	5	Other	1	Other	16	Other	14	Other	13

Pediatricians suggested more than twice the number of diagnostic labels for scenes featuring nocturnal symptoms (n = 42) and for the scene featuring a severe exacerbation (n = 29) compared to the scene depicting exercise-induced wheeze (n = 11). Overall, there was a strong negative correlation between the number of labels of suggested for a scene and the proportion of practitioners who suggested asthma as a diagnostic label, though this trend was not significant (ρ = −0.75, p = 0.14).

### Symptoms and label association

Table 3 summarizes the association between identification of the featured symptom(s) and use of the diagnostic label of asthma. We observed the strongest association (symptom identified and label of asthma suggested) for scenes with wheeze at rest and wheeze with exercise (87% and 86%, respectively); this was significantly higher than the association for scenes with nighttime wheeze and nighttime cough (64% and 75%, respectively; difference from Scene 2 to Scene 4: p<0.0001.) The largest category for Scene 5 (dyspnea and wheeze) was suggestion of the label of asthma without report of the symptoms (71%). The odds ratio for use of the asthma label given symptom identification was positive and significant for Scene 1 (OR: 11.6, 95% CI: 3.6, 36.9), Scene 2, (OR: 22.3, 95% CI: 5.0, 99.5), and Scene 3 (OR: 3.1, 95% CI: 1.9, 5.0). For Scene 4, all participants identified the symptom of cough, but only 75% suggested the label of asthma. With regard to Scene 5, there was a positive but non-significant association between symptom identification (both wheeze and dyspnea) and asthma label (OR: 1.6, 95% CI: 0.2, 10.1). Overall, the odds that practitioners who described the symptoms featured in the scenes would suggest the label of asthma as a possible cause of the presentations were 80% greater than the odds of those who did not report the primary symptoms of the scene (OR: 1.8, 95% CI: 1.1–3.0.).

**Table 3:** Observed association between identification of primary symptom(s) and suggestion of label of asthma, by scene.

Scene No.	Scene Description	n	Primary Symptom identified/asthma suggested (%)	Primary Symptom identified/asthma not suggested (%)	Primary Symptom not identified/asthma suggested (%)	Primary Symptom not identified/asthma not suggested (%)
1	Wheeze at rest	113	86.7	3.5	5.3	4.2
2	Wheeze with exercise	110	86.4	1.8	6.4	5.5
3	Nocturnal wheeze	109	64.2	18.4	5.5	11.9
4	Nocturnal cough	109	75.2	24.8	0.0	0.0
5	Dyspnea and wheeze	109	4.6	0.9	70.6	23.9

N reflects number of respondents to each question.

### Factors associated with use of the asthma label

A total of 109 (96%) respondents provided a diagnostic label(s) for all five scenes, and were included in the multivariable analysis. Overall, 54 respondents (49%) suggested the label of asthma for all five video scenes. All demographic, training and practice characteristics that were associated with suggestion of the label of asthma for all five scenes at p<0.15 are presented in Table 4. In adjusted analysis, age of the participant was negatively and significantly related to suggestion of the label of asthma for all five scenes (aOR: 0.95, 95% CI: 0.91, 0.99). Practicing in Milwaukee County was inversely related to the odds of suggestion of an asthma label for all five scenes (aOR: 0.23, 95% CI: 0.11, 0.78). We found no other significant relationships among other measured demographic characteristics (sex, race/ethnicity), training (specialization or board certification), or patient mix.

**Table 4:** Demographic, patient, and practice characteristics of participants suggesting label of asthma for all 5 vignettes (n = 54).

	Crude OR	95% CI	Adjusted OR	95% CI
General Pediatricspractice	0.50	(0.22, 1.16)	0.86	(0.33. 2.24)
Practice in a medicalschool	2.43	(0.98, 6.05)	2.15	(0.78, 5.93)
Practice in UrbanCenter	0.50	(0.22, 1.11)	0.23	(0.11, 0.78)
Age*	0.96	(0.93, 1.00)	0.95	(0.91, 0.99)

Variables were included in the analysis if chisq test p<0.15 in bivariate analysis.

* Age included in the model as a continuous variable; all other variables entered into model as binary.

## Discussion

Although asthma is the most common chronic disease that pediatricians diagnose and treat, it remains a complex clinical challenge. The disease is characterized by a variety of common phenotypes and an evolving clinical nomenclature; persistently high rates of underdiagnosis have been reported worldwide. Despite these problems, there have been few systematic attempts to understand the extent to which practicing pediatricians vary in their interpretation and labeling of the disease and its primary symptoms. Since asthma surveillance relies frequently on self- or parental-report of physician diagnosis, understanding the diagnostic patterns of physicians for respiratory disease is a critical and overlooked aspect of asthma epidemiology.

This analysis demonstrates that a sample of practicing pediatricians varied in the labels suggested to describe the likely causes of standardized presentations of asthma. Overall, there a high proportion of clinicians suggested a diagnostic label of asthma for each scene; of those clinicians who fully completed the survey, over half suggested a diagnostic label of asthma for all five scenes. Some of this variation resulted from differences in the identification of the primary characteristic of asthma featured in the scenes.

Our results also suggest that some pediatricians may be less aware of the relationship between nocturnal symptoms and asthma. Scenes that depicted nocturnal cough and wheeze had significantly less symptom-label congruence compared to the two scenes depicting wheeze at rest and wheeze with exercise. However, as evidenced in the scene that depicted wheeze and dyspnea, participants suggested the label of asthma even when they did not report the primary characteristic of asthma depicted in the scene.

Collection of demographic and practice characteristics allowed us to investigate the influence of contextual factors on symptom recognition and labeling. Age was inversely related to labeling of all five scenes as asthma; practice in Milwaukee was also inversely related to labeling. This finding is slightly counterintuitive, as urban centers in the United States generally have the highest prevalence of asthma. [21] However, given that pediatric asthma prevalence and emergency department visit rate in the US is highest among black children [22] and that black children are disproportionately impacted by asthma-related morbidity in Milwaukee [23], the absence of black individuals in the ISAAC vignettes may have contributed to this finding. Practice in a medical school was positively related to labeling of asthma in all five scenes, but did not reach statistical significance, most likely due to a limited sample size. Despite the identification of several factors that lead to asthma labeling, there is much unexplained variability in the recognition of the primary characteristics and in the labels suggested to describe these scenes.

The variability in the perception and interpretation of asthma symptoms among this group of practitioners is consistent with previous studies reporting that clinicians often disagree on the presence or absence of asthma-related respiratory signs, [24]–[26] [24]–[26] and vary in the preferred terms for descriptions of lung sounds in asthma. [9] In a general study of respiratory symptoms, Spiteri *et al.* found that practitioners who differed from their colleagues in the observation of a clinical sign were more likely to make inaccurate diagnoses. [26] Specific to asthma, Baker *et al.* found pediatric asthma specialists used different information in reaching their diagnoses and varied significantly when classifying the severity of standardized descriptions of patients with asthma. [16] In addition, symptoms such as cough have been shown to have changing clinical relevance to a diagnosis of asthma [24], and there is known variability in the value of symptom combinations to predict clinical diagnosis. [27] The current study also suggests that more attention to the variable nomenclature for asthma may be warranted.

This pilot study illustrates how standardized audiovisual presentations may be used to systematically assess and evaluate activities to raise the quality of care for asthma. In particular, our results suggest that surveys of practitioners using audiovisual simulations offer a methodology to assess how practitioners diagnose and treat all persons with asthma, even those they may not recognize or consider as having the disease. In addition, the effects of unobservable differences among physicians in the labeling of symptoms or disease can be minimized and the potential for recall and information biases that may be introduced by use of the term asthma in questionnaires can be limited.

Our results suggest that by demonstrating the potential range of presentations of asthma, audiovisual materials such as the ISAAC video have potential value in the education and training of physicians. In addition to positive impressions regarding the authenticity and quality of the videos, numerous participants suggested that the videos deserved a role in the education of physicians. Many participants expressed an interest in receiving feedback on the accuracy of their responses and on the findings in general. We believe that there is an opportunity to simultaneously improve the quality of asthma management and refine epidemiological measurements and public health surveillance of asthma by matching and co-evolving these types of audiovisual instruments with physician expectations and practices.

The results of our study are subject to several limitations. In particular, the current study reports the perceptions of a convenience sample of practitioners recruited from an email list; the group was not intended to be a representative sample of the population of practicing pediatricians in Wisconsin. The aim of this research was not to evaluate the clinical skills of pediatricians in the community, but to access a diverse sample of participants in order to assess the level of variability in perception, interpretation and labeling of common asthma symptoms that may have a bearing on diagnosis, management, and epidemiological patterns. Nevertheless, the response rate to the survey was low and may have resulted in participation by a select group. While this study used the presentations of asthma symptoms depicted in the ISAAC video, it is also important to note that these scenes were designed and validated for use as an epidemiological questionnaire. It is likely that better targeted instruments could be developed specifically for the purpose of training physicians or assessing clinical practices. Physicians generally rely on a different and more sustained set of inputs, including a characteristic history of patient-reported symptoms, one or more physical examinations, and physiological measurements, to reach a clinical diagnosis. We do not intend to suggest that the video methodology described in this report represents or simulates the general process of clinical diagnosis. With regard to clinical diagnosis of asthma, several studies have reported issues with parental understanding or report of the term “wheeze.” [28], [29] A study conducted in an ethnically and racially diverse setting reported that parents were able to better recognize audiovisual presentation of “wheeze” than to report the appropriate terminology [30]), which may have implications for provision of symptom history and accurate diagnoses, particularly for pediatric populations.

Three clinicians who had reported familiarity with the video had 87% recognition of the principal characteristics in each of the scenes and suggested asthma labels for 100% of the scenes. Despite their familiarity, they provided multiple disease labels in addition to asthma, possibly indicating that physicians cannot provide a definitive diagnosis of asthma for all five scenes. This may be for several reasons. Respondents suggested that the individuals in the ISAAC video were older than a general pediatric population, and commented on reluctance to suggest a definitive diagnosis for adults in the scenes. Second, respondents were not asked to consider the scenes in aggregate, which may have improved the proportion who offered a conclusive disease label.

Our relatively low response rate and resulting small sample size may have obscured the importance of several covariates that could have influenced the report of asthma as a diagnostic label. Further, we did not use the terms “asthma” or “ISAAC” in any communication regarding the survey in order to reduce potential biases, though it would have been advantageous to understand respondents’ experience with asthma in their clinical practices.

### Conclusion

Practicing pediatricians vary in the terms used to label standardized symptoms of asthma. This variability suggests that existing methods of ascertaining the prevalence of asthma by inquiring about a diagnostic history of asthma may underestimate the true prevalence of the disease. In addition, the results suggest that congruence between diagnostic labels and epidemiological case definitions of asthma can no longer be assumed. Further efforts to standardize and align these two important components will result in improved research, interventions and evaluations.

## Supporting Information

Appendix S1. (DOCX)
