# Supplementary material for: Variability in the Labeling of Asthma among Pediatricians
Source: PLoS One. 2013 Apr 24;8(4):e62398. doi: 10.1371/journal.pone.0062398 (PMC3634749; doi:10.1371/journal.pone.0062398)
Supplement: Appendix S1. — (DOCX) [file pone.0062398.s001.docx]

**Variability in the labeling of asthma among pediatricians**

**Van Sickle et al.**

**Instrument: Survey of pediatricians**

Name

Email Address

Date and Time Started

Date and Time Completed

Sex

Age

Race/Ethnicity

Total number of years in practice

In what areas do you practice?

Board certification

Are you currently in a pediatric residency training program?

Present clinical setting(s)

In what setting is the majority of your practice located?

Average number of patients you see per day

Approximately what percentage of your patients are insured by Medicaid?

Approximately what percentage of your patients are uninsured?

Scene 1

Please list all of the signs and symptoms that you observed in this person:

Based on this presentation, what disease is this person most likely suffering from?

Scene 2

Please list all of the signs and symptoms that you observed in this person:

Based on this presentation, what disease is this person most likely suffering from?

Scene3

Please list all of the signs and symptoms that you observed in this person:

Based on this presentation, what disease is this person most likely suffering from?

Scene 4

Please list all of the signs and symptoms that you observed in this person:

Based on this presentation, what disease is this person most likely suffering from?

Scene 5

Please list all of the signs and symptoms that you observed in this person:

Based on this presentation, what disease is this person most likely suffering from?

Have you ever seen any of these video scenes before?

Please provide any comments or feedback you may have on the survey.
